# Challenges of early detection of pancreatic cancer

**DOI:** 10.1172/JCI191942

**Published:** 2025-10-15

**Authors:** Michael J. Shen, Arsia Jamali, Bryson W. Katona

**Affiliations:** Division of Gastroenterology and Hepatology, University of Pennsylvania Perelman School of Medicine, Philadelphia, Pennsylvania, USA.

## Abstract

Pancreatic cancer (PC) is a devastating disease, due in part to its diagnosis frequently being made at an advanced stage. Ongoing efforts are aimed at identifying early-stage PC in high-risk individuals, as early detection leads to downstaging of PC and improvements in survival. However, there are a myriad of challenges that arise when trying to optimize PC early detection strategies, including selection of the appropriate high-risk individuals and selection of the test or combination of tests that should be performed. Here, we discuss the populations that are the strongest candidates for PC screening and review professional PC screening guidelines. We also summarize the current state of imaging techniques for early detection of PC and further review many studied biomarkers — ranging from nucleic acid targets, proteins, and the microbiome — to highlight the current state of the field and the challenges that remain in the years to come.

## Introduction

Pancreatic cancer (PC) is the 12th most common newly diagnosed cancer worldwide with the sixth highest mortality rate (1). Concerningly, the age-adjusted incidence rate of PC has increased among both sexes and across multiple races and ethnicities (2). PC mortality is disproportionately driven by regional and distant disease at the time of diagnosis, with 5-year survival rates of 16% and 3%, respectively (2). In contrast, localized disease has a 5-year relative survival rate of 44%, with stage 1A disease having a 5-year survival rate of more than 80% (3), highlighting the critical importance of early detection. Unfortunately, localized disease accounts for fewer than 1 of 7 PC cases at the time of diagnosis (2).

Understanding the mechanisms of pancreatic carcinogenesis is key to developing and improving approaches to PC early detection. Extensive work has been performed to elucidate the cell of origin of pancreatic ductal adenocarcinoma (PDAC), the most common type of PC (4, 5). Though these tumors have a histologically ductal appearance, evidence from genetically engineered mouse models has suggested an acinar origin proceeding through ductal metaplasia. PDAC carcinogenesis arises through one of two distinct pathways. The more frequent path involves pancreatic intraepithelial neoplasia (PanIN) as a microscopic precursor lesion that can progress from low-grade to high-grade to invasive adenocarcinoma. The less frequent pathway involves cystic pancreatic lesions, including intraductal papillary mucinous neoplasms (IPMNs) and mucinous cystic neoplasms (MCNs). Early detection efforts to date have focused on identifying early-stage PDACs (stage 1A/B) and/or precursor lesions with higher potential for progression (i.e., IPMNs with high-grade dysplasia [HGD]). Because the time from the first initiating mutation to the birth of a nonmetastatic founder cell has been estimated to be over a decade in PC (6), there may be additional opportunities to detect premalignant cells as well.

**Figure 1:**
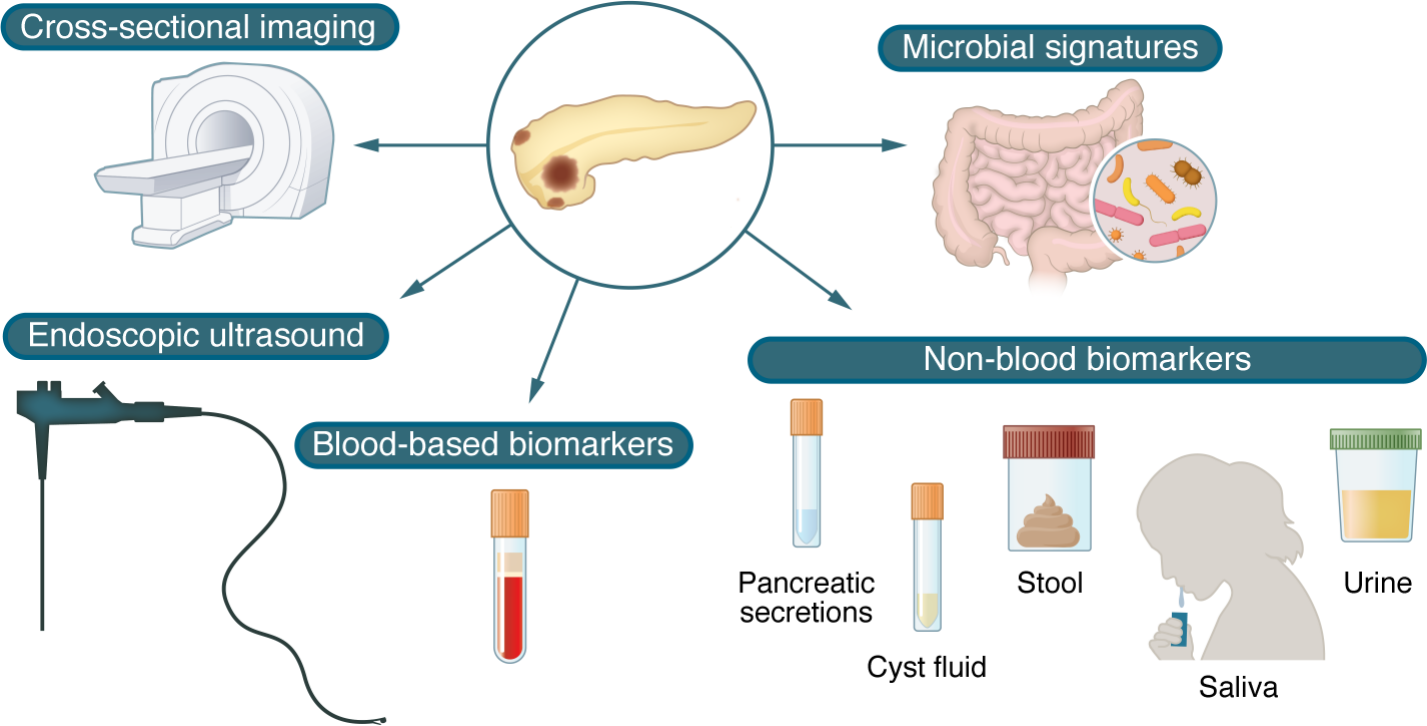
Current and investigational methods of screening for PC.

In this Review, we focus on the challenges associated with early detection of PC, highlight the populations in which early detection strategies should be considered, and describe current methods utilized for PC early detection and the future directions of research (Figure 1).

## Factors associated with an increased risk of PC

Recognizing factors associated with an increased PC risk is important for identifying the appropriate high-risk populations of individuals who may benefit from early detection strategies. Relevant factors include genetic predisposition, lifestyle risk factors, and clinical metrics, as described in Table 1.

In addition to analyses of single-factor risk contribution to PC risk, machine learning (ML) has been applied to large datasets to determine the potential to predict future PC occurrence. The applications of ML to imaging modalities and biomarkers are described below. Additionally, temporal trajectories of diagnosis codes in two large national databases have been used as inputs to artificial intelligence (AI) algorithms to predict PC occurrence (7).

## Early detection of PC in clinical practice: who, when, and how

Although the United States Preventative Services Taskforce (USPSTF) does not recommend routine screening for PC in asymptomatic adults (8), multiple professional societies and working groups have published guidance on which high-risk individuals (HRIs) should be screened, the age at which screening should start, and how screening should be performed (Table 2).

### Who.

The groups recommended for screening include those with genetic susceptibility and those with familial PC (FPC), defined as individuals having two or more directly related relatives with PC on the same side of the family, with at least one being a first-degree relative. The specific subgroups in which PC screening is recommended are outlined in Table 2. Currently, the most controversial question is whether a family history of PC is a requirement for PC screening in pathogenic germline variant (PGV) carriers, especially with respect to the hereditary breast cancer susceptibility genes *BRCA1*, *BRCA2*, *PALB2*, and *ATM*.

Although there are no formalized recommendations for screening individuals with one or more known clinical risk factors for PC, it is increasingly recognized that the presence of multiple risk factors associates with elevated cancer risk. A 2022 analysis of two large prospective cohort studies performed in the United States (9) concluded that participants with three or more risk factors (cigarette smoking, obesity, diabetes, tall height, non–type O blood group) compared with those with no risk factors was associated with a 9.24 HR for PC for those aged 60 years or younger, 3.00 for those aged 61–70 years, and 1.46 for those over age 70. Furthermore, a time-trend analysis examining the CDC National Program of Cancer Registries database from 2001 through 2018 (10) showed an increasing incidence of PC diagnoses overall and, in particular, a greater increase in the age-adjusted incidence rate for younger women (under age 55 years) than for younger men. A better understanding of whether there exist sex-specific risk factors that may underpin this difference would be important, as current screening guidelines do not differ between the sexes. Collectively, these data underscore a potential benefit to considering the clinical and demographic variables beyond PGVs and family history when deciding whom to screen for PC.

### When.

The age to start screening is dependent on the specific PC risk gene and/or the age of onset of PC in the family. Individuals with FPC or who harbor PGVs in most PC risk genes are recommended to commence screening at age 50 or 10 years prior to the earliest age of onset of PC in a relative, whichever comes first. However, for those with PGVs in *CDKN2A* and *PRSS1*, screening should start at age 40, whereas individuals with *STK11* PGVs are recommended to begin screening at age 35.

While guidelines generally agree on when to start screening, there is not yet a consensus for when screening should stop. A recent American Gastroenterological Association (AGA) clinical practice update suggests a practical approach to this question, advising clinicians to consider discontinuing screening once a patient is more likely to die of non-PC-related causes or has comorbidities making surgical resection of a detected PC infeasible (11).

### How.

PC screening is typically accomplished by imaging the pancreas with either abdominal MRI with IV contrast with cholangiopancreatography (MRI/MRCP) or endoscopic ultrasound (EUS), which is discussed in detail later in this Review. Imaging is typically performed at least annually, as it is estimated that the average T1-stage PC can progress to the T4 stage in a little over one year (12). Additionally, screening for new-onset diabetes is suggested by the Cancer of the Pancreas Screening (CAPS) Consortium guidelines. Current guidelines do not recommend other types of testing for PC screening.

## Outcomes of PC early detection studies

Multiple studies have sought to quantify the efficacy of early detection, i.e., whether screening in HRIs is able to (a) diagnose PC at an earlier stage and (b) confer a survival benefit, which is the gold standard for screening test effectiveness. Ideally, these outcomes would be assessed in a randomized, controlled trial, however, such a trial that would offer a “no-screening” arm is unlikely to be undertaken today.

Understanding baseline imaging findings among HRIs is an important first step. Among 1,400 HRIs enrolled in the Pancreatic Cancer Early Detection (PRECEDE) Consortium study, 35.1% had a pancreatic cyst identified, and one incidental PC (*CDKN2A* carrier, stage IIB) was found on baseline imaging (13). Age and FPC were both associated with a higher likelihood of harboring a pancreatic cyst.

However, the difficulty in detecting early-stage, high-risk pancreatic lesions was highlighted by the international CAPS Consortium (14). Of 2,500 HRIs,13 developed a new high-risk pancreatic lesion a median of 11 months after a prior screening exam, and 77% of these new lesions had progressed beyond the pancreas at the time of identification, highlighting the need for better early diagnostic tools.

A Dutch study following HRIs with an annual EUS in addition to MRI/MRCP (15) found a 9.3% cumulative incidence of PC in PGV carriers compared with 0% in noncarriers with FPC. Of those found to have malignancy, 60% of the tumors were resectable, and median survival was 18 months.

In 2022, the Cancer of Pancreas Screening-5 (CAPS5) multicenter, prospective cohort study (16) reported that 9 participants developed PC, with 78% of cancers being stage I by pathology and 89% deemed resectable. Eight participants in this study underwent surgical resection for concerning cystic lesions found on screening, with 3 (38%) found to have HGD and 5 (63%) found to have low-grade dysplasia, illustrating a downside of screening, which may result in surgical resection of benign lesions. However, this study did not demonstrate significant surgical complications.

A subsequent comparative cohort study compared 26 HRIs enrolled in the CAPS program who developed PC with over 1,500 matched PC patients from the SEER database (17). 38.5% of the tumors in the high-risk screening group were diagnosed at stage I, compared with 10.3% of those in matched controls, while 27.3% of the high-risk group tumors were stage T1 compared with 4.4% of the matched control tumors. Strikingly, the HRIs demonstrated higher overall survival (median 61.7 months) compared with matched controls (median 8.0 months), and higher 1- and 5-year survival probabilities (84% and 50% vs. 38% and 9%).

In summary, there is emerging evidence that PC screening can downstage PC and improve long-term survival among HRIs who go on to develop PC. However, substantial challenges associated with PC early detection remain, and these present opportunities to improve imaging modalities as well as to incorporate both blood and non-blood-based biomarkers into PC screening programs.

## Imaging

Imaging is the cornerstone of PC early detection strategies for HRIs, however there remains debate about which imaging modality is most effective and whether improvements could allow for earlier diagnosis of PC or PC precursor lesions.

*Which imaging modality is best*? CT of the abdomen with IV contrast (CT), MRI/MRCP, and EUS are the most studied PC screening imaging modalities. In 2012, the CAPS3 investigators screened 216 asymptomatic HRIs with CT, MRI/MRCP, and EUS, which visualized 14%, 77%, and 79% of all detected lesions, respectively (18). Concordance between EUS and MRI/MRCP was highest, and MRI/MRCP and EUS each detected considerably more pancreatic lesions (mostly cysts) than did CT.

In a 2016 multicenter prospective study of 139 asymptomatic HRIs in the Netherlands (19), who underwent simultaneous EUS and MRI/MRCP, 11 clinically relevant lesions were identified, with 6 (55%) detected by both modalities. MRI/MRCP was more sensitive for cystic lesions, while EUS detected 2 solid lesions not seen on MRI/MRCP (one of which was found to be an early-stage PC). This was supported by a 2022 study from the Netherlands, where EUS was superior to MRI/MRCP for the detection of solid lesions, whereas MRI/MRCP outperformed EUS in detecting cystic lesions, primarily driven by increased detection of sub-centimeter-sized cysts (15).

A 2022 single-center prospective study examining concordance of baseline EUS and MRI/MRCP found 62.3% concordance, fair strength of agreement in identifying any pancreatic abnormality, very good strength of agreement in identifying a dilated main pancreatic duct, and very high concordance with respect to worrisome lesions, with one pancreatic neuroendocrine tumor missed by MRI/MRCP (20).

While MRI/MRCP is the imaging study typically used for PC screening, in Li-Fraumeni syndrome (LFS), whole-body MRI (wbMRI) is instead used for pan-cancer screening. Recent work demonstrated that wbMRI identifies pancreatic lesions at lower rates compared with dedicated abdominal MRI/MRCP and therefore should not be used as the sole PC screening modality in HRIs (21).

Together, these data demonstrate that EUS and MRI/MRCP are the preferred imaging modalities for PC early detection, with EUS being superior at solid lesion detection, while MRI/MRCP is superior at cystic lesion detection, especially sub-centimeter-sized cysts. Future research in this field and more consistency in clinical decision making will require an increase in the standardization of imaging reporting for HRIs. In 2022, the PRECEDE consortium released minimal MRI/MRCP sequence recommendations and proposed a standardized reporting template for both MRI/MRCP (22) and EUS (23).

### Advances in imaging technology.

Over the past decade, there has been substantial interest in using ML and AI to enhance the diagnostic utility of pancreatic imaging studies. A 2022 study (24) evaluated four ML classifiers trained on a dataset of volumetric pancreas segmentations from patients with PC with prediagnostic contrast-enhanced CT scans along with matched controls. The best-performing of these models (support vector machine) correctly classified 95% of prediagnostic and 90% of control CT scans in the test set and showed a higher discrimination performance than did two radiologist readers.

In 2023, a deep learning algorithm was trained on over 3000 noncontrast CT scans to identify PCs and seven subtypes of non-PC lesions, with an AUC over 0.98 for lesion detection when applied to a multicenter validation dataset of over 6,000 patients (25). The algorithm significantly outperformed human readers, even when readers were provided contrast-enhanced CT scans. When the algorithm diagnosis probabilities were used as an aid to human readers, their performance identifying PCs was significantly improved. The authors additionally showed an ability to identify PCs on noncontrast chest CTs. With further real-world validation, such tools may be a way to utilize common imaging studies obtained for non-PC reasons to inform the selection of individuals for further assessment.

AI is also being applied to both improve operator performance in obtaining EUS images as well as aid in their interpretation. A single-center, randomized, controlled trial in China used a deep learning–based AI device to identify anatomic EUS stations (26). Endoscopists using this device missed fewer stations compared with endoscopists without AI. Multiple groups have also demonstrated the use of AI for integration of clinical data and EUS images to assist in the diagnosis of both solid (27) and cystic (28, 29) pancreatic lesions.

Identifying high-grade PanINs on imaging would likely improve early diagnosis of PC, as these precursor lesions are currently not detectable on standard pancreatic imaging. Recent work in genetically engineered mouse models and ex vivo human pancreas specimens focused on using specific MRI sequences to identify PanIN-associated changes (30), although this work has yet to be validated in vivo in humans. Multiple studies have also examined whether PC is preceded by nonpancreatic imaging changes. One study quantified adipose tissue and skeletal muscle up to 5 years prior to PC diagnosis, finding adipose tissue wasting in the 6 months prior to PC diagnosis and earlier skeletal muscle loss in individuals at the time of diagnosis (31). A separate group examined both imaging and metabolic findings in the 36 months prior to PC diagnosis (32), finding decreases in visceral and subcutaneous adipose tissue near the time of diagnosis.

As imaging technologies and protocols advance, the high-quality data generated will enable application of emerging technologies, such as AI-assisted image analysis, to increase diagnostic sensitivity by identifying lesions that cannot currently be consistently visualized either by direct visualization or by inferring their effects on surrounding tissue. However, refinement will be needed to avoid unnecessary tissue sampling and surgical procedures due to the potential for false-positive results.

## Biomarkers

Extensive research has been undertaken to develop PC markers that can be used alongside imaging or, ideally, to eventually replace imaging and/or risk-stratify HRIs who need imaging. Development of a robust biomarker requires multiple phases including discovery, development, and validation of a clinical assay, prospective studies, and, finally, assessment in clinical trials (33). Existing biomarker studies have numerous limitations, such as relying on samples collected at the time of PC diagnosis to identify biomarkers, failure to characterize pre-PC biomarker trajectories, and lack of validation in appropriately powered independent cohorts. Currently, the guidelines do not consistently recommend the regular use of any biomarkers for HRIs, however, ongoing research holds promise for future advances (34).

## Blood-based biomarkers

### Blood glucose/hemoglobin A1c.

Hyperglycemia may be detected up to 36 months before PC diagnosis (35). In fact, every 10 mg/dL increase in fasting blood glucose (FBG) is associated with a 14% increase in the rate of PC (36). Therefore, monitoring FBG and/or hemoglobin A1c (HbA1c) can be considered in HRIs. However, there is no consensus or guidance about how to change clinical management on the basis of changes in FBG and HbA1c, or what level of change should prompt concern. Furthermore, prediabetes and diabetes are increasingly diagnosed in the general population, therefore, this approach may not offer the appropriate specificity as a PC screening test. A recent study of HRIs undergoing screening over a median follow-up of 41 months showed no difference between newly diagnosed PC and controls in the frequency of elevated glucose and HbA1c levels (37). However, larger studies with extended follow-up are warranted prior to drawing firm conclusions.

### Carbohydrate antigen 19-9.

Carbohydrate antigen 19-9 (CA19-9, also known as sialyl Lewis A), is the most extensively studied biomarker of PC. Apart from PC, this cell-surface glycoprotein can be elevated in various benign and malignant conditions of the pancreas and extrapancreatic tissues (i.e., stomach, biliary system, salivary glands) (38–45). Serum CA19-9 levels encompass 79%–81% sensitivity and 82%–90% specificity for the detection of PC in asymptomatic individuals, however, given the low incidence of PC, the positive predictive value (PPV) of serum CA19-9 remains inadequate, ranging from approximately 0.5% to 0.9%, which restricts its application as a universal screening tool for PC (46). Serial CA19-9 measurement may facilitate the early detection of PC, since CA19-9 levels may increase up to two years prior to PC diagnosis, with 50% sensitivity and 99% specificity for early-stage disease 0–6 months before diagnosis (47).

**Figure 2:**
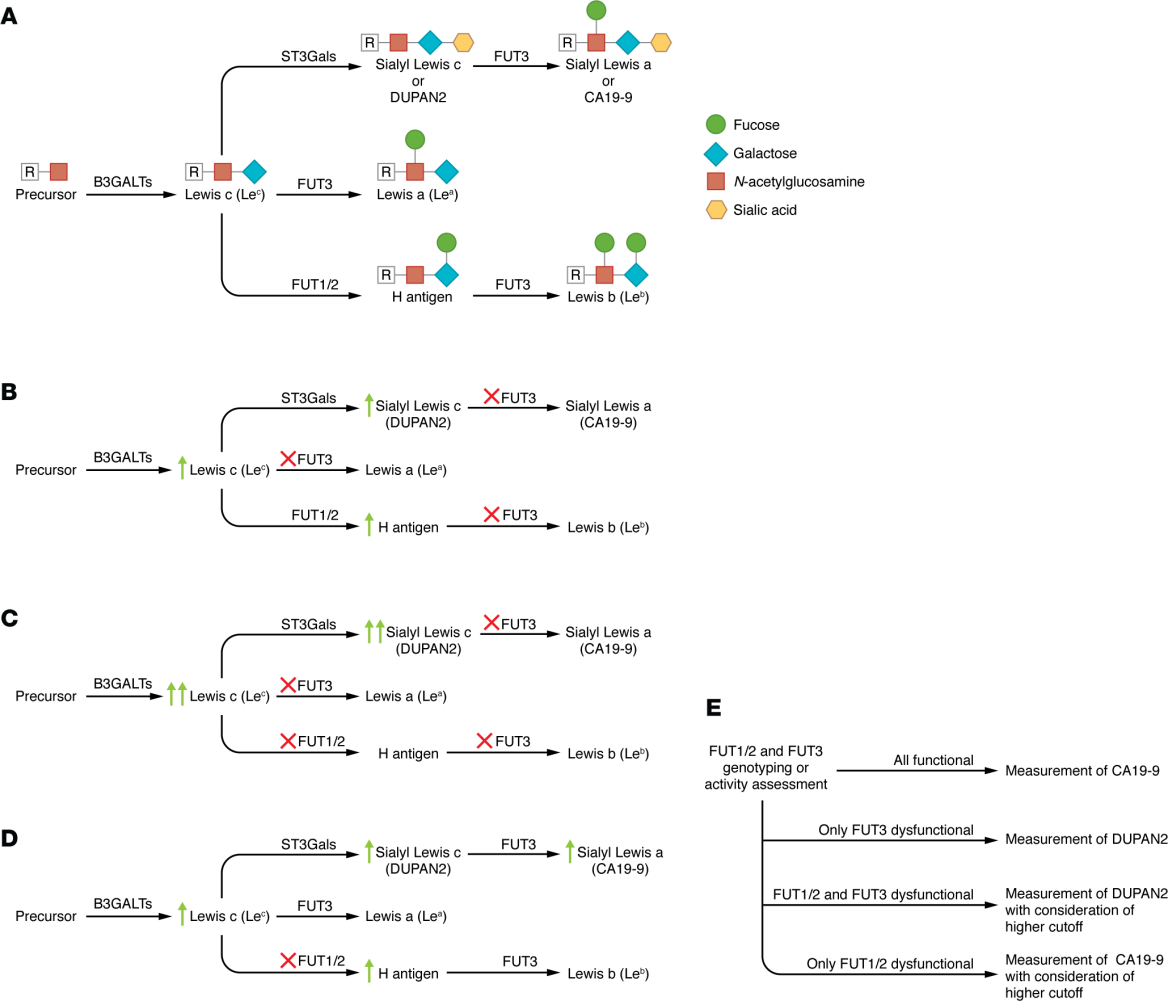
CA19-9 biosynthesis. (**A**) The first step of biosynthesis of CA19-9 is the addition of a galactose molecule to type 1 precursor chains, catalyzed by β1,3-galactosyltransferases (B3GALTs) to yield Lewis c, which itself serves as the precursor for Lewis a, Lewis b, and CA19-9 (sialyl Lewis a). For biosynthesis of CA19-9, β-galactoside α2-3-sialyltransferases (ST3Gals) catalyze the addition of a sialic residue to the 2,3 linkage of the GlcNAc moiety to yield sialyl Lewis c, which is also called DUPAN2. Next, a fucose residue is added at the 1,4-residue of the GlcNAc moiety of sialyl Lewis c (DUPAN2), which is solely catalyzed through the enzymatic activity of α1,3/4-fucosyltransferase (FUT3) to produce CA19-9. In addition to serving as the precursor of CA19-9, Lewis c may undergo other biochemical alterations via either (i) FUT3 to produce Lewis a or (ii) subsequent activities of α1,2-fucosyltransferase (FUT1/2) and subsequently FUT3 to produce Lewis b. (**B**) Lack of FUT3 activity leads to Lewis blood type negative (Le^a–b–^), lack of production of CA19-9, and accumulation of DUPAN2. (**C**) Lack of both FUT1/2 and FUT3 activity leads to higher levels of DUPAN2 compared with lack of FUT3 activity alone. (**D**) Lack of FUT1/2 activity along with intact FUT3 activity increases CA19-9 levels. (**E**) Schematic algorithm for the measurement of CA19-9 and DUPAN2 for early detection of PC.

As depicted in Figure 2A, the biosynthesis of CA19-9 involves multiple enzymatic steps, shared with the synthesis of Lewis blood group antigens (48–50). CA19-9 levels are considerably affected by alterations in the expression of these enzymes (Figure 2, B–D) (51–53). Importantly, approximately 10% of the population does not produce CA19-9 (51, 54, 55), and that rate is higher in some vulnerable populations (56, 57), which can lead to false-negative results. An algorithm for individualized measurement considerations for CA19-9 or its precursor Duke pancreatic monoclonal antigen type 2 (DUPAN2) is demonstrated in Figure 2E.

### Other protein biomarkers, multiplex arrays, and proteomics.

Separate from CA19-9, there has been extensive study of other blood-based proteins involved in various aspects of development of PC including proliferation, invasion, and interaction with surrounding normal tissue and the immune system. Comprehensive appraisal of each of these targets is beyond the scope of the current Review, however, a concise summary of the most widely examined proteins reviewed in meta-analyses is provided in Table 3.

The combination of multiple protein biomarkers in multiplex assays or proteomics approaches may offer greater diagnostic accuracy compared with a single biomarker alone. A multicenter study showed that measurement of up to 10 targets was sufficient for robust discrimination of PC (58). A separate study identified a signature of 29 biomarkers capable of distinguishing early-stage PC with an AUC of 0.96 (95% CI: 0.94–0.98), 93% sensitivity, and 95% specificity (59).

The first commercially available protein-based blood test for PC early detection was IMMray PanCan-d, a 9-plex biomarker signature consisting of CA19-9 and 8 protein targets (60). IMMray PanCan-d was designed to return a positive, borderline, negative, or test-not-performed (for CA19-9 nonexpressers [≤2.5U/mL]) result, and could separate controls from early-stage PC with 89% sensitivity and 99% specificity (60). The assay was first offered in 2021, however, it was voluntarily discontinued in 2023. In HRIs undergoing pancreatic screening, IMMray PanCan-d exhibited a robust negative predictive value of 99% (with an estimated PC prevalence of 2%), however, its PPV was suboptimal and dependent on interpretation of borderline test results (61). An updated assay from the same company based on 4 protein biomarkers became commercially available in September 2025 as PancreaSure, however prospective data evaluating this test are still needed (62).

Furthermore, recently, a single peptide substrate probe was developed into PAC-MANN, a magnetic nanosensor assay with a fluorescent probe to distinguish PC from controls with an AUC of 0.91 in a training dataset. Although the test demonstrated low sensitivity for early PC with sensitivity of 62% and 56% for stage I and stage II PC, respectively, addition of CA19-9 considerably improved the sensitivity to 85% and 81% for stage I and stage II PC, respectively (63).

### Cell-free circulating tumor DNA.

Assessment of circulating tumor DNA (ctDNA), which are small fragments of DNA shed from apoptotic tumor cells into the bloodstream, is a promising approach for detecting cancer (64–68), although it may be limited by low levels of ctDNA in early-stage disease as well as by contamination from noncancerous DNA.

Individuals with PC have higher ctDNA methylation than do individuals without cancer and individuals with acute or chronic pancreatitis (69). Early ctDNA studies focused on limited genes, with a model consisting of the methylation status of *BMP3*, *RASSF1A*, *BNC1*, *MESTv2*, *TFPI2*, *APC*, *SFRP1*, and *SFRP2* showing an AUC of 0.86 (sensitivity of 76% and specificity of 83%) (69). Further attempts to improve the diagnostic accuracy revealed that in early-stage PC, the AUC for the methylation status of *ADAMTS1* and/or *BNC1* was 0.95 (95% CI: 0.90–0.98) (70). Aiming to interrogate genome-wide methylation, whole-genome bisulfite sequencing revealed robust specificity (99.3%, 95% CI: 98.3%–99.8%), with sensitivity reaching 67.3% (95% CI: 60.7%–73.3%) for stages I–III in 12 prespecified cancers (including PC) (71). A subsequent study optimized the test, reproduced these findings, and highlighted an accuracy of 88.7% (95% CI: 87.0%–90.2%) in predicting the cancer’s tissue of origin among true positives, however, sensitivity for PC remained relatively low for early stages (61.9% and 60.0% for stages I and II, respectively), although higher sensitivities were observed for more advanced stages (85.7% and 95.5% for stages III and IV, respectively) (72). These findings supported the development of the commercial Galleri test for multicancer detection, however, the performance of Galleri in HRIs is not reported (73).

The epigenetic alteration 5-hydroxymethylcytosine in ctDNA can be used to detect various cancers, including PC (74). A regression model utilizing 37 genes with the most significant increase in 5-hydroxymethylcytosine showed promising performance in identifying PC with an AUC of 0.92 in a training dataset and AUCs of 0.92 and 0.94 in external validation datasets (75). In a similar study, an initial comparison of hydroxymethylation patterns in PC tissue versus normal tissue led to the identification of 366 genes with decreased and 43 genes with increased hydroxymethylation. Via measurement of the hydroxymethylation status of these genes in ctDNA, an algorithm was developed after matching for BMI, age, and smoking status, and showed a sensitivity of 68.3% (95% CI: 51.9%–81.9%) and an overall specificity of 96.9% (95% CI: 96.1%–97.7%) for detection of early-stage PC (76). This led to the development of the Avantect Pancreatic Cancer Test, for which a recent validation study showed 68.3% sensitivity (95% CI: 51.9%–81.9%) and 96.9% specificity (95% CI: 96.0%–97.6%) in a validation dataset (77).

Combining ctDNA with other blood-based biomarkers may further improve PC diagnostic accuracy. Detecting *KRAS* gene mutations in ctDNA via the Safe-Sequencing System, a sensitive technology that minimizes errors in massively parallel sequencing, along with four protein biomarkers — CA19-9, carcinoembryonic antigen (CEA), osteopontin (OPN), and hepatocyte growth factor (HGF) — yields 64% sensitivity and 99.5% specificity for stage I/II PC (66). Advancing this approach, CancerSEEK was developed to measure the levels of eight protein biomarkers (CA-125, CA19-9, CEA, OPN, HGF, prolactin, myeloperoxidase, and tissue inhibitor of metalloproteinases 1 protein) alongside the presence of mutations in 1,933 distinct genomic positions in ctDNA. The median sensitivity of CancerSEEK was approximately 70% for detecting the eight most common cancer types (including PC) in stages I–III, with greater than 99% specificity. Specifically, for PC, the tested achieved 72% sensitivity, and ML algorithms could correctly identify PC as the most likely cancer type in 81% of individuals with positive test results (78).

SeekInCare is another multicancer detection test that combines protein biomarkers and ctDNA and is currently being studied. Preliminary findings suggest an 82.4% sensitivity for PC (79).

### Cell-free miRNAs.

miRNAs are 7 to 25 nucleotide-long noncoding RNAs that posttranscriptionally regulate genes, and several miRNAs play key roles in PC biology (80–82). While various miRNAs are significantly increased in PC, one study showed that among 7 miRNAs, only miR-16 and miR-196a independently distinguished PC. Addition of CA19-9 to the model increased the accuracy with an AUC of 0.98, a sensitivity of 92.0%, and a specificity of 95.6% (83).

Another study identified 38 potentially differentially expressed miRNAs in PC and developed 2 indices: index I (4 miRNAs: miR-145, -150, -223, and -636) and index II (10 miRNAs: miR-26b, -34a, -122, -126*, -145, -150, -223, -505, -636, and -885.5p). Index I demonstrated an AUC of 0.86 (95% CI: 0.82–0.90) with 85% sensitivity (95% CI: 79%–90%) and 64% specificity (95% CI: 57%–71%), whereas index II improved the AUC to 0.93 (95% CI: 0.90–0.96) with 85% sensitivity (95% CI: 79%–90%) and 85% specificity (95% CI: 80%–85%) for detecting early PC, with further improvement noted with the addition of CA19-9 (84). In another study using next-generation miRNA-Seq, 100 highly expressed miRNAs were selected to create a diagnostic model for detection of PC via ML, showing an AUC of 0.94 (95% CI: 0.91–0.97) to separate PC from controls, with further improvement noted with CA19-9 addition (85).

In a similar attempt, a multi-gene, amplification-based detection system was implemented to simultaneously analyze and select 135 miRNAs to build the OncoSweep classifier, a proprietary prediction algorithm for cancer classification. OncoSweep could identify various types of cancers with 91.4% sensitivity and 85% specificity, although PC-specific data were not available (86). In 2024, It was announced that the OncoSweep Pancreas Spotlight will be available at the City of Hope Cancer Center (Duarte, California, USA) for PC screening in HRIs.

miRNA-Seq has also led to the identification of various small miRNA panels that may have utility in early diagnosis of PC. A triple panel of hsa-miR-1246, -205-5p, and -191-5p could distinguish PC with an accuracy ranging from 79.0% to 83.5% (87). Similarly, a triple-miRNA signature (let-7i-5p, miR-130a-3p, and miR-221-3p) could discriminate healthy controls from stage I PC with an AUC of 0.97 (95% CI: 0.91–1.0), and from stage II PC with an AUC of 0.97 (95% CI: 0.95–0.99), which could be further enhanced by addition of CA19-9 (88). Nevertheless, the lack of consistency between the identified target miRNAs in these studies needs to be further explored.

### Exosomes.

Exosomes play an important role in communication among and between cancer cells and the surrounding milieu by transferring proteins and nucleic acids. Exosomal miRNAs are generally more stable and may carry more tumor-specific information compared with their unpackaged counterparts, which are passively released by apoptotic cells into the circulation. In a large multicenter study (89), RNA-Seq was performed on stage I/II PC, identifying cell-free and exosomal miRNAs leading to a refined panel of miRNAs. In combination with CA19-9 levels, the panel showed a robust accuracy in detecting early-stage PC with an AUC of 0.99 (95% CI: 0.98–1.00) with 93% sensitivity (95% CI: 88%–98%). In a prospective validation study of participants from Japan, the United States, South Korea, and China, there was consistent accuracy across the cohorts with an AUC of 0.97, 95% sensitivity, and 96% specificity in the US cohort (90).

Beyond miRNAs, analysis of other exosomal contents may further facilitate early detection of PC. For instance, glypican 1, a cell-surface proteoglycan, was identified by mass spectrometry as a specific protein enriched in exosomes derived from PC cells that might be used in distinguishing healthy individuals from those with PC (91). However, glypican 1 expression was not replicated in exosomes of some PC cell lines (92). Other exosomal proteins are currently being studied (92–94).

In summary, in addition to CA19-9, cell-free genetic material and miRNA demonstrate a potential for early detection of PC. Nevertheless, further studies are paramount to assess their accuracy and cost-effectiveness as clinical screening tools.

## Pancreatic secretion analysis

Evaluating pancreatic secretions may enhance the specificity of diagnostic biomarkers since their levels avoid nonspecific blood alterations influenced by other tissues or extrapancreatic pathology (95, 96). Nevertheless, assessment of pancreatic secretions is technically cumbersome, requiring endoscopy with duodenal aspiration or endoscopic retrograde cholangiopancretatography (ERCP) with pancreatic duct cannulation and immediate processing of collected pancreatic secretions to maintain sample integrity. Furthermore, the use of secretin to enhance the sample yield may lead to side effects. Nonetheless, studies of pancreatic secretion cytology, protein, DNA, and extracellular vesicles have been conducted and are summarized in Table 4.

## Cyst fluid analysis

Given the heterogeneity of pancreatic cysts, various biochemical, protein, RNA, and DNA biomarkers have been investigated to characterize the cyst type, particularly in differentiating between serous and mucinous cysts. Here, we focus on the markers used to identify high-risk cysts, including those with HGD and PC with cystic features.

Among gene targets for diagnosing high-risk cysts, *KRAS* and *GNAS* are the most widely studied. A meta-analysis showed that, while *KRAS* and *GNAS* have high specificity for differentiating serous cysts from mucinous cysts, they performed poorly in differentiating HGD or PC from low-grade dysplasia. Specifically, *KRAS* and *GNAS* mutations showed a pooled specificity of 35% (95% CI: 31%–40%) and 51% (95% CI: 28%–75%), respectively, in detecting HGD and PC (97). Mutations in other DNA targets including in *CDKN2A*, *PIK3CA*, *SMAD4*, and *TP53* showed poor sensitivity, but a high specificity of at least 95% (97–99).

Given the low sensitivity and specificity of testing individual genetic biomarkers, next-generation sequencing has been used to improve pancreatic cyst fluid analysis. The combination of *KRAS* and *GNAS* mutations and alterations in *TP53*, *PIK3CA*, and *PTEN* showed 89% sensitivity and 100% specificity for HGD and invasive adenocarcinoma in IPMNs (98). Building on the testing of multiple DNA targets, PathFinderTG was developed (100–102) and uses the quality and quantity of DNA, *KRAS*, and *GNAS* mutations and the loss of heterozygosity in multiple loci associated with tumor suppressor genes (*VHL*, *OGG1*, *PTEN*, *MXI1*, *TP53*, *SMAD4*, *DCC*, *CDKN2A*, *CDKN2B*, *RNF43*, *NME1*, *PSEN2*, *TFF1*, *RUNX3*, *CMM1*, *LMYC*, *MCC*, *APC*, *NF2*). The findings are reported as PanDNA (molecular analysis only) or PancraGEN, which integrates these data with clinical information to offer a comprehensive cyst risk assessment (low-risk [benign and statistically indolent] vs. high-risk [statistically higher risk and aggressive]). Comparison of PancraGEN with Fukuoka/Sendai 2012 criteria for risk stratification of pancreatic cysts in cases with negative cytology assessment for malignancy showed comparable sensitivity and a negative predictive value, but significantly higher specificity, PPV, and a positive likelihood ratio for PancraGEN (103).

The DNA- and RNA-based PancreaSeq test, developed by the University of Pittsburgh (Pittsburgh, Pennsylvania, USA), initially evaluated 74 PC-related genetic alterations to identify high-risk cysts. Next, the test was narrowed to assess alterations in gene or mRNA expression of *KRAS*, *GNAS*, *BRAF*, *TP53*, *PRKACA/B*, *ALK*, *NTRK1/3*, *RET*, *SMAD4*, and *CEACAM5* to determine whether a cyst was neoplastic. PancreaSeq showed 82% sensitivity and 100% specificity in detecting advanced HGD and early cancer in pancreatic cysts. Of note, the test provides information on cyst type and the risk of progression to high-grade dysplasia or cancer (104, 105) and is currently implemented in select institutes (106, 107).

Among protein biomarkers in clinical practice, carcinoembryonic antigen (CEA) is well appreciated for the differentiation of mucinous (higher CEA) and serous (lower CEA) cysts. However, CEA performs poorly in distinguishing HGD or cancer from low-grade dysplasia, with 40% sensitivity (95% CI: 34%–47%) and 71% specificity (95% CI: 66%–75%) (97). mAB-Das1, a murine monoclonal antibody that reacts with precancerous and cancerous esophageal, gastric, and pancreatic epithelium, showed a more promising ability to detect high-risk cysts including invasive carcinoma, HGD, or intestinal-type IPMNs with intermediate-grade dysplasia, with 88.2% sensitivity (95% CI: 0.78%–0.95%) and 99.0% specificity (95% CI: 0.95%–1.00%) (108). Targeted mass spectrometry indicated MUC5AC as another protein biomarker with relatively high sensitivity and specificity for detecting HGD and cancer (109). MUC5AC in conjugation with prostate stem cell antigen yielded an overall accuracy of 94% in the validation cohort.

Other protein biomarkers studied for the detection of high-risk cysts, including IL-1β, PGE2, tripeptidyl peptidase 1, and telomerase activity, exhibit lower sensitivities or specificities (97, 110–113). VEGF-A has been proposed as a marker for benign cysts (114, 115). However, studies are limited on its role in determining the malignancy risk in mucinous cysts.

Another recent study showed that a 10-feature multiomics panel consisting of serum and cyst biomarkers — including S100A8, LGALS3, SNORA66, miR-216b-5p, IGHV3-72, IGJ, IGHA1, PPBP, miR-3180, and miR-3180-3p — could identify HRIs with an AUC of 0.97. Despite promising findings, a lack of definitive pathologic evaluation of the cysts limits the generalizability of the findings (116).

## Urine

Urine analysis offers a convenient and noninvasive potential method for PC detection, however, the accuracy of a urine test may be affected by conditions that alter kidney function and urine composition such as kidney disease, proteinuria, hematuria, and bacteriuria. A summary of urine biomarkers is presented in Table 5. Despite the promising findings, the accuracy of these tests remains insufficient for regular use in HRIs undergoing PC screening (117).

## Saliva

Saliva serves as a noninvasive and safe source of proteins, metabolites, and miRNAs, making it a promising candidate for PC detection (Table 5). In addition, oral microbiota have also been explored. Despite initial promising findings, oral microbiota lack reproducibility across different populations. For instance, bacterial biomarkers for *N*. *elongata* and *S*. *mitis* showed an AUC of 0.90 for discriminating individuals with PC from healthy controls (118). However, another study showed that individuals with PC were more commonly found to host *Streptococcus* and *Leptotrichina* and are less commonly positive for *Veillonella* and *Neisseria* in saliva (119). Although saliva-based biomarkers demonstrate a potential for PC detection, further studies are necessary to improve their accuracy and ensure reproducibility across populations.

## Stool

Stool may contain tumor-associated protein and genetic material that can be used to detect cancer (Table 5). Furthermore, the gut microbiome may also serve as a novel avenue for early detection of PC. Individuals with PC have reduced gut microbial diversity, an increased abundance of potential pathogens, and a decreased abundance of beneficial bacteria (120). Combination of 40 genera associated with PC achieved an AUC of 0.84 (95% CI: 0.78–0.91) with 86% sensitivity and 67% specificity (120). A study from Israel showed differences in 14 bacterial features that could differentiate PC from controls (AUC = 0.82) (121), while in a Spanish cohort, fecal metagenomic classifiers based on 27 microbial species could differentiate PC with an AUC of 0.84, which was further improved to 0.94 with addition of CA19-9. Validation in a German population produced comparable results (122). Nevertheless, despite the promising findings, inclusion of fecal microbiota for PC screening is currently limited by factors that affect microbiota composition including age, diet, antibiotics use, physical activity, geographic location, psychological status (123), inconsistencies in identified species across studies (120–122), high inter-subject variability, and a weak association of altered microbiota with precancerous lesions (121).

## Cost-effectiveness

PC screening can downstage PC diagnosis and prolong survival after diagnosis, however, whether screening is cost-effective is debatable. Given the low rate of conversion to PC of HRIs undergoing screening, two meta-analyses have estimated that at least 111–135 individuals need to be screened to identify a single high-risk lesion (124, 125). A recent review (126) examined 8 cost-effectiveness studies of HRIs, most of which found that PC screening could be cost-effective, with the caveat that the risk thresholds for cost-effective screening were higher than the lifetime risks for some PC risk genes. Challenges identified by this analysis include heterogeneity in inputs and negative consequences of increased screening, such as more false-positives and potentially unnecessary surgical procedures.

A tiered approach to screening has been posited by groups such as the Early Detection Research Network (127, 128). This approach would first identify individuals with clinical predictors of elevated PC risk, subject them to biomarker-based screening, and then assess those with positive biomarkers using imaging, which could further improve cost-effectiveness.

## Future directions and conclusions

**Figure 3:**
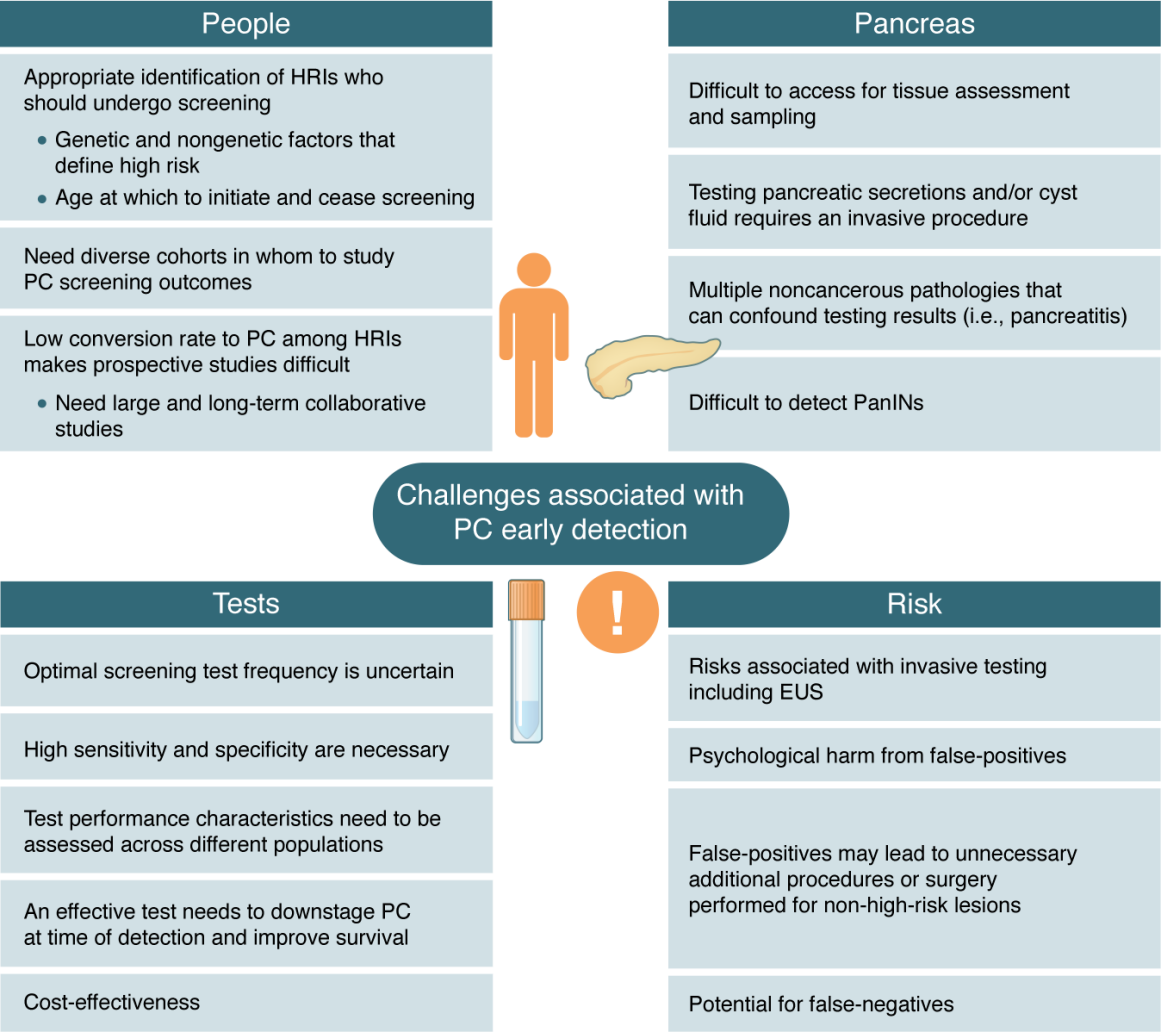
Challenges associated with early detection of PC.

While the goal of improving early detection of PC has been notably advanced over the past two decades, there remain many areas where iterative improvements can better identify individuals with early-stage PC or high-risk premalignant lesions. Continued accumulation of data encompassing single-gene PGVs, polygenic risk scores, and lifestyle risk factors may help refine the HRIs eligible for PC screening and the optimal initiation age and interval between screening tests. Figure 3 summarizes select current challenges in the PC early detection field.

The choice of imaging modality continues to evolve, as data hint at distinct benefits with both MRI and EUS. The application of AI to existing imaging studies may also allow for extraction of findings beyond the perception of humans and perhaps mine imaging performed for other indications to obtain clues portending the future development of PC. Although not yet regularly used clinically, biomarkers from blood, pancreatic secretions, cyst fluid, urine, saliva, and stool may individually, or in combination, add another layer for risk stratification. However, biomarkers need high sensitivity and specificity, and dedicated prospective studies of promising biomarkers are needed to validate their effectiveness.

Furthermore, screening regimens should not be considered independent of cost, especially in settings limited by patient resources and/or availability of equipment and expertise, therefore, the development of cost-effective approaches is important. To advance the field of PC early detection, large international groups such as the CAPS research program and the PRECEDE Consortium will be necessary to enroll enough patients to adequately power PC early detection studies. The efforts of these groups, along with the many researchers working to better understand the biology of PC, may drive a shift toward improved outcomes for this challenging disease.

## Figures and Tables

**Table 3:**
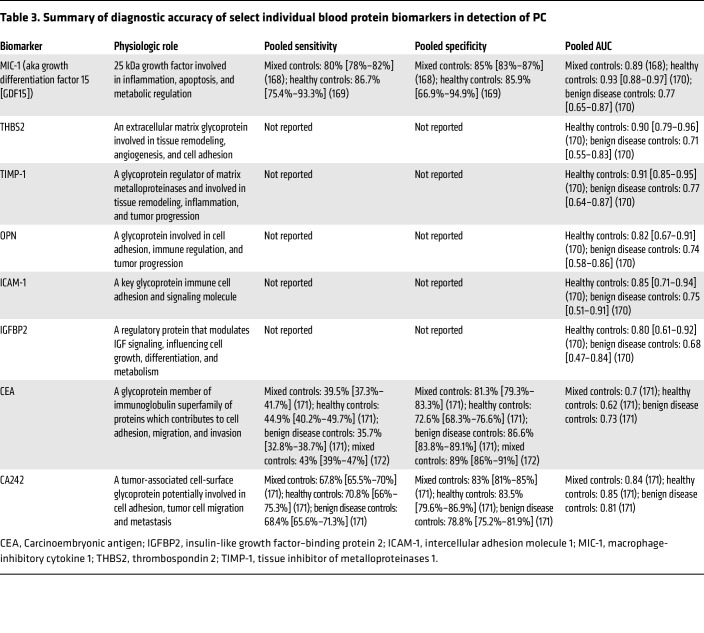
Summary of diagnostic accuracy of select individual blood protein biomarkers in detection of PC

**Table 2:**
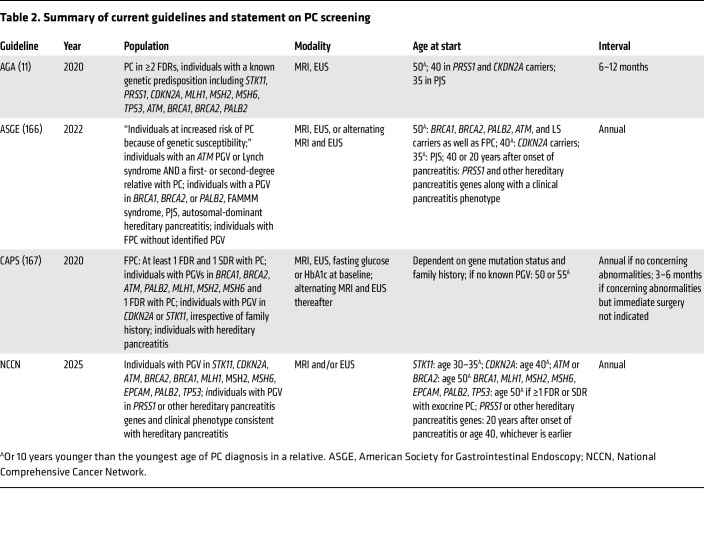
Summary of current guidelines and statement on PC screening

**Table 1:**
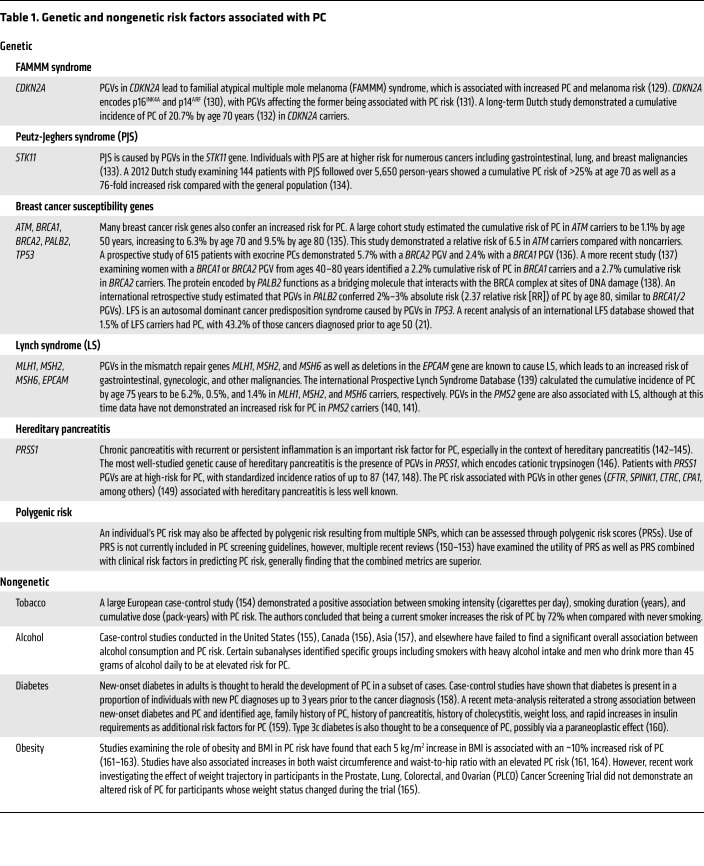
Genetic and nongenetic risk factors associated with PC

**Table 5:**
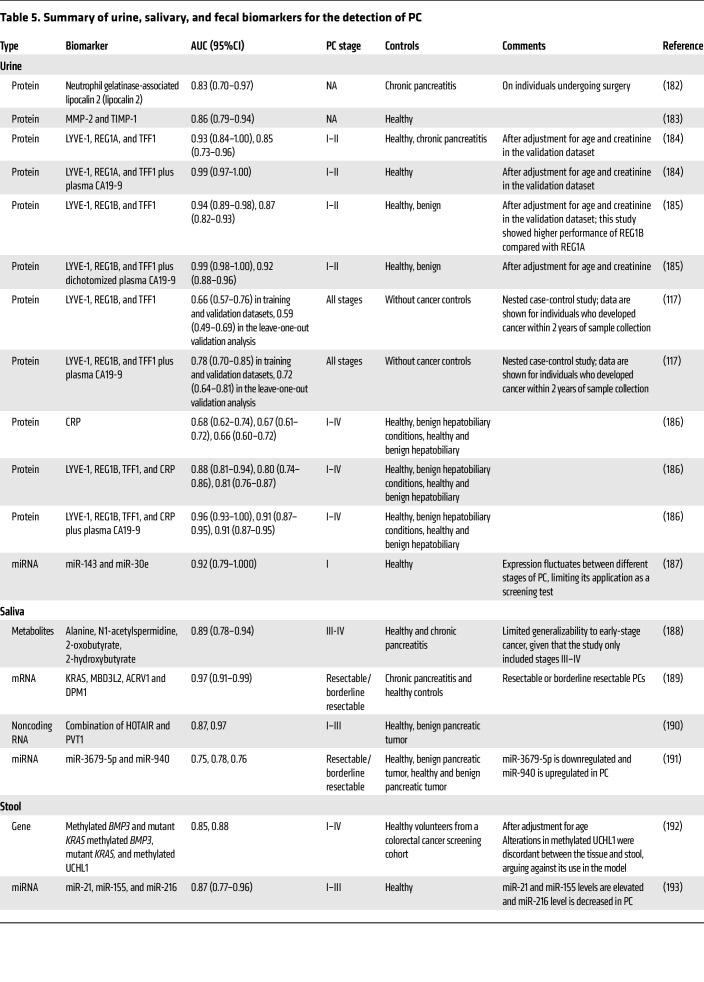
Summary of urine, salivary, and fecal biomarkers for the detection of PC

**Table 4:**
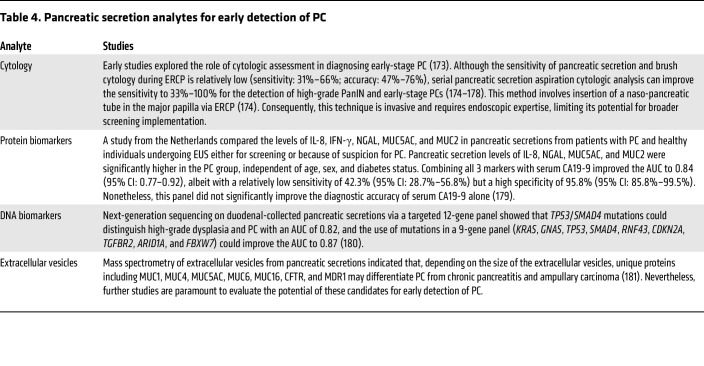
Pancreatic secretion analytes for early detection of PC

## Version 1 10/15/2025

Electronic publication
